# ACADEMIC DETAILING INTERVENTION: INCREASING TRENDS OF NALOXONE PRESCRIBING IN RURAL ARKANSAS

**DOI:** 10.1093/geroni/igae098.2844

**Published:** 2024-12-31

**Authors:** Leah Tobey-Moore, Meghan Breckling, Mohab Ali, Robin McAtee

**Affiliations:** University of Arkansas for Medical Sciences, Little Rock, Arkansas, United States; University of Arkansas for Medical Sciences, Little Rock, Arkansas, United States; University of Arkansas for Medical Sciences, Little Rock, Arkansas, United States; University of Arkansas for Medical Sciences, Little Rock, Arkansas, United States

## Abstract

This longitudinal study analyzes naloxone prescribing trends of rural primary care providers (PCPs) in Arkansas (AR). As one of the HRSA-funded Geriatric Workforce Enhancement grant recipients, the AR Geriatric Education Collaborative (AGEC) worked with a rural federally qualified healthcare system (ARcare) to reduce the likelihood of patient’s accidental overdose by co-prescribing naloxone, when indicated. A multi-disciplinary Academic Detailing (AD) team of a PT, PharmD and MBBChB conducted AD trainings. AD is clinician-to-clinician customized education connecting recipients to updated evidence and practice guidelines. These interactive telehealth trainings stimulated PCPs to share knowledge deficits and perspectives, encouraged a commitment to increase patient safety and adhere to naloxone prescribing guidelines from Centers for Disease Control and state laws especially during cases when naloxone should be co-prescribed alongside high-risk medications like opioids. ARcare PCPs received “Preventing Opioid Overdose with Naloxone” AD training. Before training, previous naloxone prescribers (group 1) prescribed naloxone 39 times, 106 times in period 1 (P1=11 months post-training), and 91 times in period 2 (P2=22 months post-training), a 14.2% decrease. Group 2 previously did not prescribe naloxone during the baseline period, then prescribed 46 times in P1 and 63 times in P2, a 40% increase. Group 3 did not prescribe naloxone in P1, but prescribed naloxone 13 times during P2. Overall, P1 data from all groups totaled 152 naloxone prescriptions after AD, a 290% increase; in P2, naloxone was prescribed 167 times, a 328% increase. In summary, AD was effective in encouraging naloxone prescribing among prescribers in rural AR.

